# Diagnosis and Surveillance of Neonatal Infections by Metagenomic Next-Generation Sequencing

**DOI:** 10.3389/fmicb.2022.855988

**Published:** 2022-03-24

**Authors:** Rong Zhang, Yan Zhuang, Zheng-hui Xiao, Cai-yun Li, Fan Zhang, Wei-qing Huang, Min Zhang, Xiao-Ming Peng, Chao Liu

**Affiliations:** ^1^Department of Neonatology, Hunan Children’s Hospital, Changsha, China; ^2^Department of Emergency, Hunan Children’s Hospital, Changsha, China; ^3^Department of Medical, Hangzhou Matridx Biotechnology Co., Ltd., Hangzhou, China

**Keywords:** neonatal infections, metagenomic next-generation sequencing, *Mycobacterium tuberculosis*, *Legionella pneumophila*, *Bacillus cereus*

## Abstract

Microbial infections cause significant morbidity and mortality in neonates. Metagenomic next-generation sequencing is a hypothesis-free and culture-free test that enables broad identification of pathogens and antimicrobial resistance genes directly from clinical samples within 24 h. In this study, we used mNGS for etiological diagnosis and monitoring the efficacy of antibiotic treatment in a cohort of neonatal patients with severe infections. The median age was 19.5 (3–52) days, median gestational age was 37.96 (31–40^+3^) weeks, and the median birth weight was 3,261 (1,300–4,300) g. The types of infectious diseases included pneumonia, sepsis, and meningitis. mNGS reported microbial findings in all cases, which led to changes in antibiotic treatment. These included cases of *Mycobacterium tuberculosis*, *Legionella pneumophila*, and *Bacillus cereus*. Eight of ten infants recovered after antibiotic adjustment and showed normal development during follow-up. On the other hand, neurological retardation was seen in two infants with meningitis. mNGS enabled etiological diagnosis and guided antibiotic therapy when all conventional methods failed to discover the culprit. It has the potential to cut down the overall cost and burden of disease management in neonatal infections.

## Introduction

Infectious disease is an important cause of morbidity and mortality in neonatal populations (Cailes et al., 2018). Seventeen deaths per 100 live births and 6,700 neonatal deaths per day were reported in 2019 at a global scale (Gu et al., 2019). A total of 48% of neonatal death older than 7 days can be attributed to microbial infections, which is the main cause of death during this period (Collaborators, 2015; Khan et al., 2017). Conventional microbiological tests usually have long turn-around time and low sensitivity (Singh et al., 2021).

The gold standard for microbial identification is culture. However, several factors contributed to the low detection rate of culture in neonatal infections, including limited sample volume, the presence of intermittent bacteremia, and maternal intrapartum antimicrobial exposure. The detection rate of culture ranged from 3–10.29% in early-onset sepsis (EOS) to 16.7–33% in late-onset sepsis (LOS; Abdelhamid, 2017; Marks et al., 2020; Huggard et al., 2021). Other commonly used culture-independent tests such as blood cell count and the immature to total neutrophils (I/T ratio) can be used to infer bacterial infection (Shane et al., 2017). The inflammatory serum markers including C-reactive protein (CRP), Procalcitonin (PCT), and cytokines (interleukin 6, interleukin 8, tumor necrosis factor α, etc.) can also be used to indicate microbial infections, albeit with low specificity (Sharma et al., 2018; Brown et al., 2019). With the advances in molecular biology, polymerase chain reaction (PCR) and 16S/18S rRNA sequencing have been developed, which had limited scope of targets (Cummings et al., 2016; Kosacki et al., 2020). Therefore, an accurate diagnostic test for broad pathogen detection is needed.

Metagenomic Next-Generation Sequencing (mNGS) is culture-independent, which unbiasedly sequences both host and microbial nucleic acids extracted from a variety of clinical specimens (Deurenberg et al., 2017; Miller et al., 2019; Pohl et al., 2019; Wilson et al., 2019; Li et al., 2020). It has the advantage of detecting a broad range of pathogens and antibiotic resistance genes (Gu et al., 2019; Mitchell and Simner, 2019). Although the current mNGS technology has shown several advantages, there are still challenges for the clinical application: (1) the amount of non-microbial DNA presents in clinical samples (mostly derived from human cells) negatively impact the diagnostic sensitivity of microorganisms; (2) microbial read count cannot be compared between samples, making it unlikely to assess the changes in pathogen load if multiple mNGS tests were carried out for the same patient (Gu et al., 2019). Taken the above-mentioned concerns into consideration, we have developed a quantitative metagenomic sequencing approach (Zhou et al., 2021) to monitor the cellularity and microbial abundance of each sample, which was achieved by including a nucleic acid internal control (IC) spiked at constant concentrations into all specimens. In addition to monitoring the host level, we could also use the ratio of microbial reads/spike reads to quantify the abundance of microorganisms. Different microbes have genomes of varying length and GC content. Therefore, it was not likely to directly extrapolate the copy number or CFU based on this ratio. However, this ratio was independent of host nucleic acid and thus might be better than read count (RPM) to represent the pathogen load. In this study, we conducted the quantitative metagenomic sequencing for neonatal patients with severe infections in which conventional methods failed to elucidate the etiology. We reported 10 cases that were successfully and treated according to mNGS.

## Materials and Methods

### Ethics Statement

This study was performed in compliance with relevant laws and institutional guidelines and was approved by Ethics Committee of Hunan Children’s Hospital (HCHLL-2021-71). Written informed consent was obtained from all patients or their legal guardians.

### Patients and Samples

Ten subjects suspected of microbial infections in Neonatal Intensive Care Unit (NICU) were recruited. Fresh specimens including peripheral blood, sputum, bronchoalveolar lavage fluid (BALF), and cerebrospinal fluid were collected for mNGS testing and conventional microbiology tests including bacterial/mycoplasma/chlamydia/fungal culture, antigen test for influenza A/B virus, parainfluenza virus, adenovirus respiratory syncytial virus and antibody test for cytomegalovirus, herpes simplex virus, toxoplasma, and rubella virus in each patient.

### Preparation of Spike-in Internal Control

The internal control (IC) DNA was synthesized, amplified by PCR (TAKARA PrimeSTAR^®^ HS DNA Polymerase, Cat# R044), and purified using magnetic beads (Matridx, Cat# MD012). All procedures were performed inside a Biosafety cabinet. Qubit fluorometric quantitation was performed on the amplicons (Thermo Fisher, Qubit^™^ dsDNA HS Assay Kit, Cat# Q32854). The nucleotide sequences of IC DNA can be found in a previous study (Zhou et al., 2021). ICs were added to each sample prior to nucleic acid extraction at a final concentration of 0.02 ng/μl.

### Library Preparation and Sequencing

Whole blood was centrifuged at 1,600 g for 10 min and supernatant was centrifuged at 16,000 g for 10 min to separate plasma. For sputum, BALF and cerebrospinal fluid, DNA, or RNA sequencing were performed. DNA or RNA sequencing PCR-free library preparation was prepared by reverse transcription (for RNA), enzymatic fragmentation (except for plasma), end repairing, terminal adenylation, and adaptor ligation (NGSmaster^™^ library preparation, Matridx, Cat# MAR002; Luan et al., 2021). Concentration of libraries was quantified by real-time PCR (KAPA) and pooled. Shotgun sequencing was carried out on illumina Nextseq platform. Approximately 20 million of 75 bp single-end reads were generated for each library. For each run, one negative control (artificial plasma mixed with fragmented human genomic DNA) and one positive control (a mixture of inactivated bacteria, fungi, and pseudoviral particles containing synthesized DNA or RNA fragments of adenovirus and influenza A virus, respectively) were included for quality control.

### Bioinformatic Pipeline

Raw sequencing data were analyzed by a bioinformatic pipeline, which included the following steps: (1) unnecessary adapter sequences and low-quality bases (Q-score cutoff, 20) were trimmed of in the pipeline; (2) human host sequences were eliminated by mapping to the human reference genome (GRCh38.p13) using BWA (BurrowsWheeler alignment)^1^; and (3) after removal of low-complexity reads, the remaining sequencing data were simultaneously aligned by BWA to reference database [NCBI nt database and GenBank (Benson et al., 2013)] in order to identify microbial species (Cantalupo and Pipas, 2019).

### mNGS Reporting Criteria

Microbial reads identified from a library were reported if: (1) the sequencing data passed quality control filters (library concentration > 50 pM, Q20 > 85%, and Q30 > 80%); (2) negative control (NC) in the same sequencing run does not contain the species or the RPM (sample) /RPM (NC) ≥ 5, which was determined empirically as a cutoff for discriminating true positives from background contaminations.

### Statistical Analysis

Patient characteristics were described using frequency, median, mean, range, and summary data with SPSS version 22.0.

## Results

### Clinical Characteristic and Overall Diagnostic Performance of mNGS

The age of the 10 participants ranged from 2 to 52 days, and 4 participants (40%) were female. The median age was 19.5 (3–52) days, median gestational age was 37.96 (31–40 + 3) weeks, and median birth weight was 3,261 (1,300–4,300) g. The types of infectious diseases included pneumonia, sepsis, and meningitis. The clinical characteristics of enrolled patients were listed in Table 1. The patient samples were analyzed by classical methods (culture, antigen test, and PCR) and mNGS. mNGS reported microbial findings in all cases, which led to changes in antibiotic treatment. These included cases of *Mycobacterium tuberculosis*, *Legionella pneumophila*, and *Bacillus cereus*. Eight of ten infants recovered after antibiotic adjustment and showed normal development. On the other hand, neurological retardation was seen in two infants with meningitis. The mNGS findings were listed in Table 2.

**Table 1:** Clinical characteristics of the 10 patients.

Patient no./Sex/Age, d	Infection data			Initial signs	RF	CF	NA	LOS in NICU, d	Final diagnosis
	CRP (mg/dl)	PCT (ng/ml)	IL-6 (pg/ml)						
1/M/19	205.08	16.44	1,387	fever, tachypnea	+	+	−	19	*Mycobacterium tuberculosis*
2/F/4	>320	1.07	181.5	fever, cough, dyspnea	+	+	−	33	*Legionella pneumophila*
3/F/2	206.68	>100	>5,000	cough, fever	+	+	−	26	*Moraxella catarrhalis Staphylococcus aureus*
4/F/38	3.74	0.16	13.46	spasmodic cough	+	−	−	11	*Chlamydia trachomatis*
5/M/52	41.84	0.17	76.92	fever, abdominal distention	+	−	−	83	*Ureaplasma parvum*
6/M/5	98.46	2.32	165.8	fever, cyanosis	+	+	+	47	*Streptococcus mitis*
7/M/23	33.06	0.31	101.0	cough, fever, tachypnea	+	−	+	43	*Streptococcus pasteuri Human betaherpesvirus 5*
8/M/7	122.59	>100	>5,000	fever, convulsion	+	+	+	40	*Escherichia coli*
9/M/3	192.9	12.18	324.1	fever, convulsion	−	−	+	26	*Streptococcus agalactiae*
10/F/15	168.1	18.12	23.06	fever, dyspnea, convulsion	+	+	+	13	*Bacillus cereus*

F, female; M, male; CRP, C-reactive protein; PCT, Procalcitonin; IL-6, cinterleukin 6; RF, Respiratory failure; CF, Circulatory failure; NA, Neurological abnormality; LOS, length of stay; and NICU, neonatal intensive care unit.

**Table 2:** mNGS findings of the 10 patients.

Patient#/gestation age, wk	Conventional method results			mNGS results								Therapy			Follow-up (Mo)/outcome
	Culture	Antigen	PCR	Sample	TAT (h)	Initial Host index	Pathogens	IR	Initial Microbial index	FR	Final Host index	Final Microbial index	Antibiotics, d	Other	
1/39	Sputum/Blood/CSF(−)	Respiratory/TORCH (−)	*EV/CA* (−)	Blood	13	28826.85	*Mtb*	59	10723.30	ND	/	/	Isoniazid/ rifampicin/pyrazinamide,67	Ventilation 7d	3/recovered
2/40	Sputum/Blood/CSF/BALF(−)	Respiratory/TORCH (−)	*EV*(−)	Blood	22.5	29143.69	*LP*	361	13653.88	NF	29485.81	NF	Erythromycin,19	Plasma transfusion Ventilation 12d	2/recovered
3/40^+3^	Sputum(*MP*)/Blood (−)	Respiratory/TORCH (−)	*EV/CA* (−)	Blood	41.7	28392.78	*Mcat*	110	10994.57	NF	21320.92	NF	Amikacin/Ceftazidime/Linezolid,24	Ventilation 3d	5/recovered
4/38^+1^	Sputum/Blood (−)	Respiratory/TORCH (−)	*EV*(−)	Sputum	40.7	26006.36	*CT*	1,330	13036.10	NF	19779.90	NF	Erythromycin,11	Salbutamol nebulization	5/recovered
5/31	Sputum/Blood (−)	TORCH (−)	*EV/CA* (−)	Sputum	21	26180.42	*UP*	65	8430.86	6	22020.46	1784.12	Erythromycin,7	Bishop’s fistula of ileum and santulli fistulaVentilation 10d PICC 30d	4/recovered
6/39^+1^	Sputum/Blood (−)	Respiratory/TORCH (−)	*EV*(−)	Sputum	18	27934.48	*S. mitis*	1,286	15876.97	NF	26254.30	NF	Oxacillin/Vancomycin,14	Repair of VSD and PFO, ligation of PDA Ventilation 10d	4/recovered
7/40^+1^	Sputum/Blood/CSF(−)	*RSV*(+)	*EV*(−)	Blood	17.7	23752.90	*S. pasteuri*& *HHV-5*	6/12	2661.86/3543.13	ND	/	/	Vancomycin/Acyclovir,43	NCPAP 4d DEX 4d	2/recovered
				CSF	17.7	31468.11	*S. pasteuri& HHV-5*	32/1	12767.09/7773.50	4/11	28348.48	7672.03/9126.95			
8/39^+5^	Sputum/blood/CSF(−)	Respiratory/TORCH(−)	*EV*(−)	blood	43.5	27536.51	*E. coli*	NF	/	ND	/	/	Meropenem/Cefotaxime,40	phenobarbitone 7d/midazolam 3d/prednisone 7d	1/recovered
				CSF	43.5	31482.51	*E. coli*	13	9777.09	NF	25699.22	NF			
9/38^+3^	Sputum/blood/CSF/stool(−)	Respiratory/TORCH(−)	*EV*(−)	blood	/	/	/	ND	/	ND	/	/	Penicillin,26	phenobarbitone 16d/midazolam 3d DEX 4d	1/encephalomalacia in left side of brain
				CSF	18.6	27271.33	*GBS*	15,611	15390.84	NF	27033.76	NF			
10/34^+2^	Sputum/blood/CSF(−)	*RSV*(±)	*EV/CA* (−)	Blood	21	32275.98	*B. cereus*	4	9508.34	ND	/	/	Gentamicin/Vancomycin,17	Ventilation 3d phenobarbitone 8d	1/diffused encephalomalacia
				CSF	21	29712.14	*B. cereus*	61	9559.24	2	24454.21	804.66			

wk, week; CSF, cerebral spinal fluid; *MP, Mycoplasma pneumonia*; TORCH, *Toxoplasma, Rubella, cytomegalovirus*, herpes, and others; Respiratory, including *influenza A/B virus, parainfluenza virus, adenovirus, and respiratory syncytial virus*; *RSV*, *respiratory syncytial virus; EV, Enterovirus*; *CA*, *Coxsackie Virus*; TAT, turn-around time; *Mtb, Mycobacterium tuberculosis*; *LP, Legionella pneumophila*; *Mcat, Moraxella catarrhalis*; *CT*, *Chlamydia trachomatis; UP, Ureaplasma parvum*; *S. mitis, Streptococcus mitis*; *S. pasteuri*, *Streptococcus pasteuri; HHV-5, Human betaherpesvirus 5; E. coli*, *Escherichia coli; GBS, Streptococcus agalactiae*; *B. cereus, Bacillus cereus*; IR, Initial reads; FR, Finial reads; NF, not found; ND, not done; PICC, peripherally inserted central catheter; VSD, ventricular septal defect; PFO, patent foramen ovale; PDA, patent ductus arteriosus; DEX, dexamethasone; and Mo, months.

### Case Descriptions

#### Mycobacterium tuberculosis

**Figure 1:**
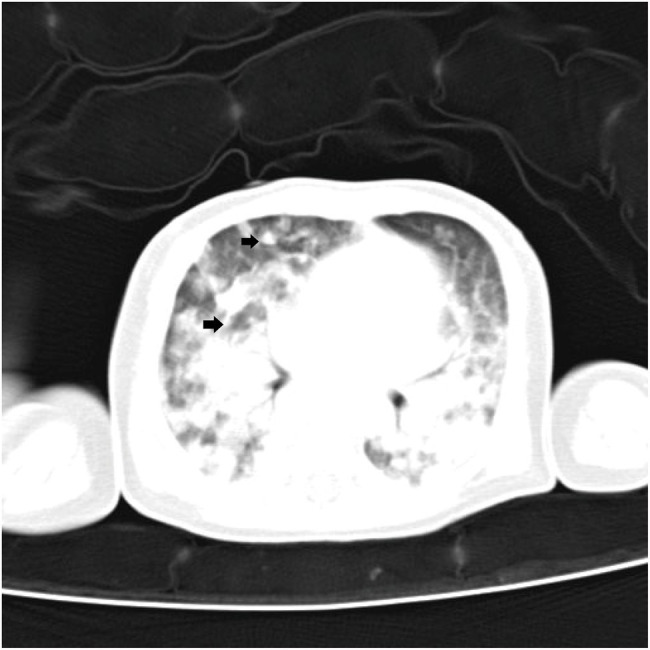
Chest computed tomography image of patient 1 showing many miliary nodules (arrowheads) can be seen in both lungs.

**Figure 2:**
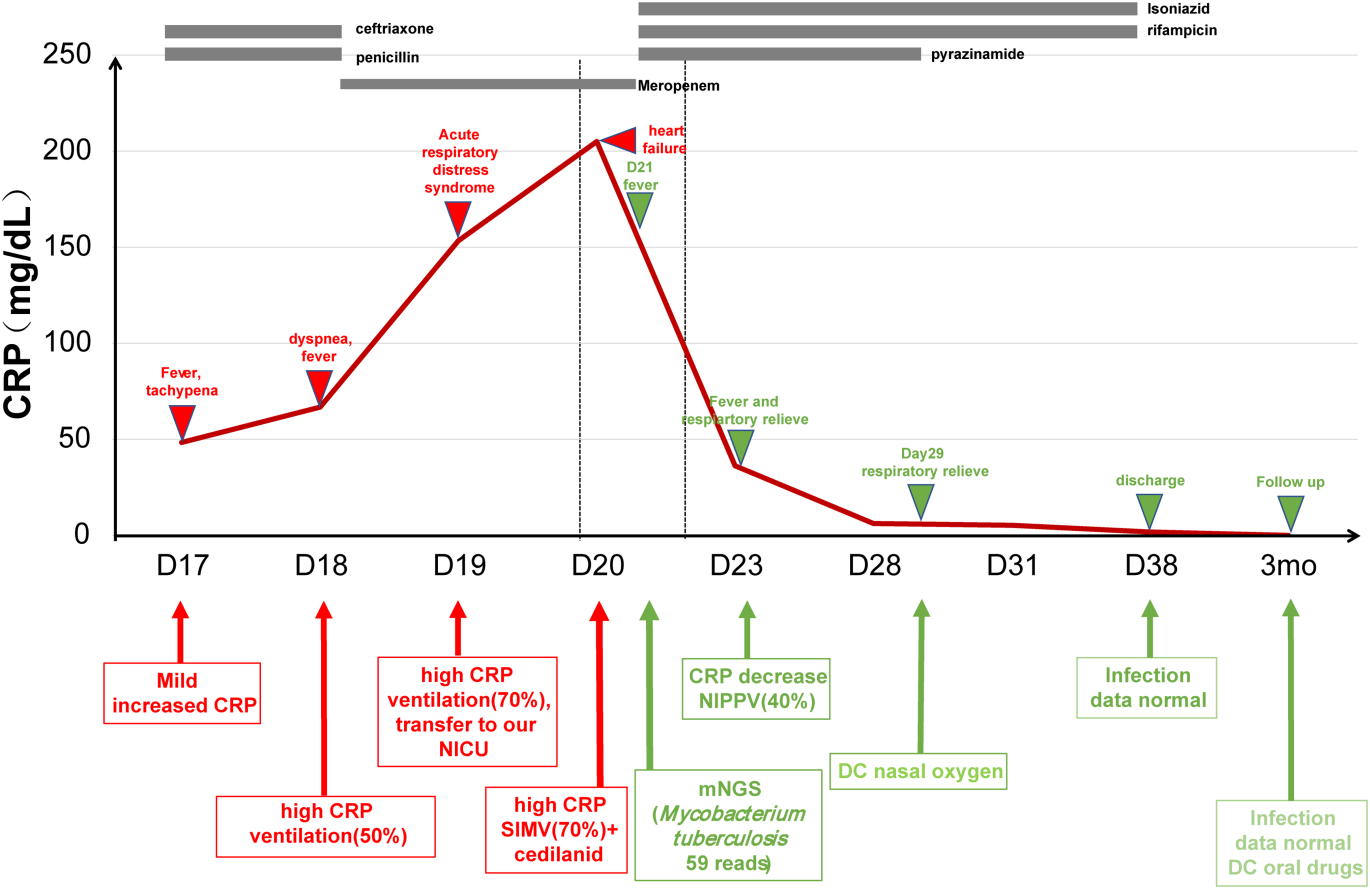
The treatment course in *Mycobacterium tuberculosis* case. CRP, C-reactive protein; NICU, neonatal intensive care unit; and NIPPV, non-invasive positive pressure ventilation.

Patient 1 (19-day-old male) was admitted due to intermittent fever and tachypnea. The patient was born by vaginal delivery to a 30-year-old gravida 4, para 1 mother at 39 weeks of gestation. The Apgar score was 10/10 at 1 and 5 min, respectively. The patient developed fever (39.5°C) and required ventilation on day 16. During a 3-day-stay at a local hospital, the patient’s symptoms rapidly deteriorated and sepsis screen reported a CRP of 153.04 mg/dl and Procalcitonin (PCT) of 16.44 ng/ml. The patient was then transferred to our hospital due to respiratory distress, which needed 70% oxygen supplement to maintain saturation after upgrading antibiotics to meropenem and vancomycin. Microbiological culture of sputum, blood, and cerebrospinal fluid were negative. The biochemical analysis of cerebrospinal fluid (CSF) was also normal. On day 21, we collected peripheral blood for mNGS testing, which reported 59 reads of *Mycobacterium tuberculosis complex*. This finding was confirmed by T.SPOT-TB test of blood and Xpert MTB/RIF assay of sputum. Chest computed tomography (CT) scan revealed many miliary nodules with different sizes, densities, and shapes which had blurred edges and partly fused into a piece in both lungs (Figure 1). The patient’s mother had abnormal CT scan that was consistent with TB infection, although with no clinical manifestations. We applied antituberculosis treatment (Isoniazid, rifampicin, and pyrazinamide) in combination with oral Isoniazid and rifampicin (Figure 2). Blood count, CRP, growth, and neurological development were normal during the 1-month follow-up.

#### Legionella pneumophila

**Figure 3:**
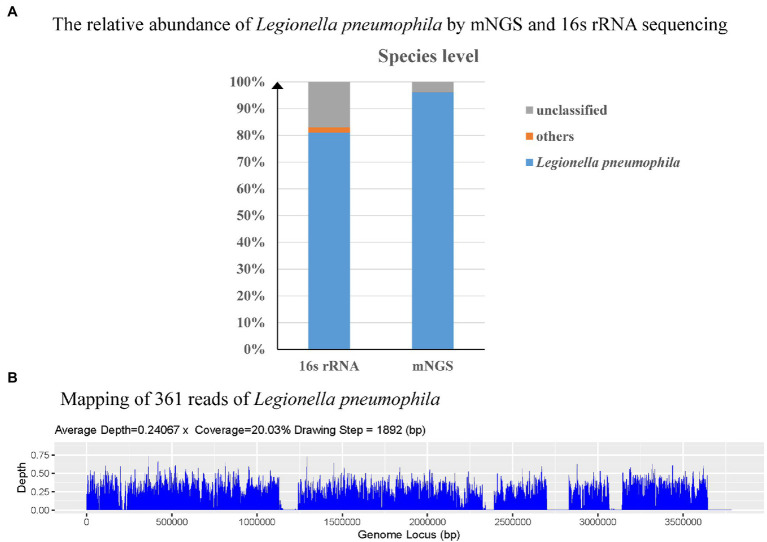
Diagnosis of *L. pneumophila* infection by means of Unbiased Next-Generation Sequencing (mNGS) and 16s ribosomal RNA (rRNA) sequencing. The relative abundance of *L. pneumophila* from the patient’s bronchoalveolar lavage fluid (BALF) by mNGS and 16s rRNA sequencing **(A)**. Sequence reads mapped to *L. pneumophila* by mNGS data **(B)**.

**Figure 4:**
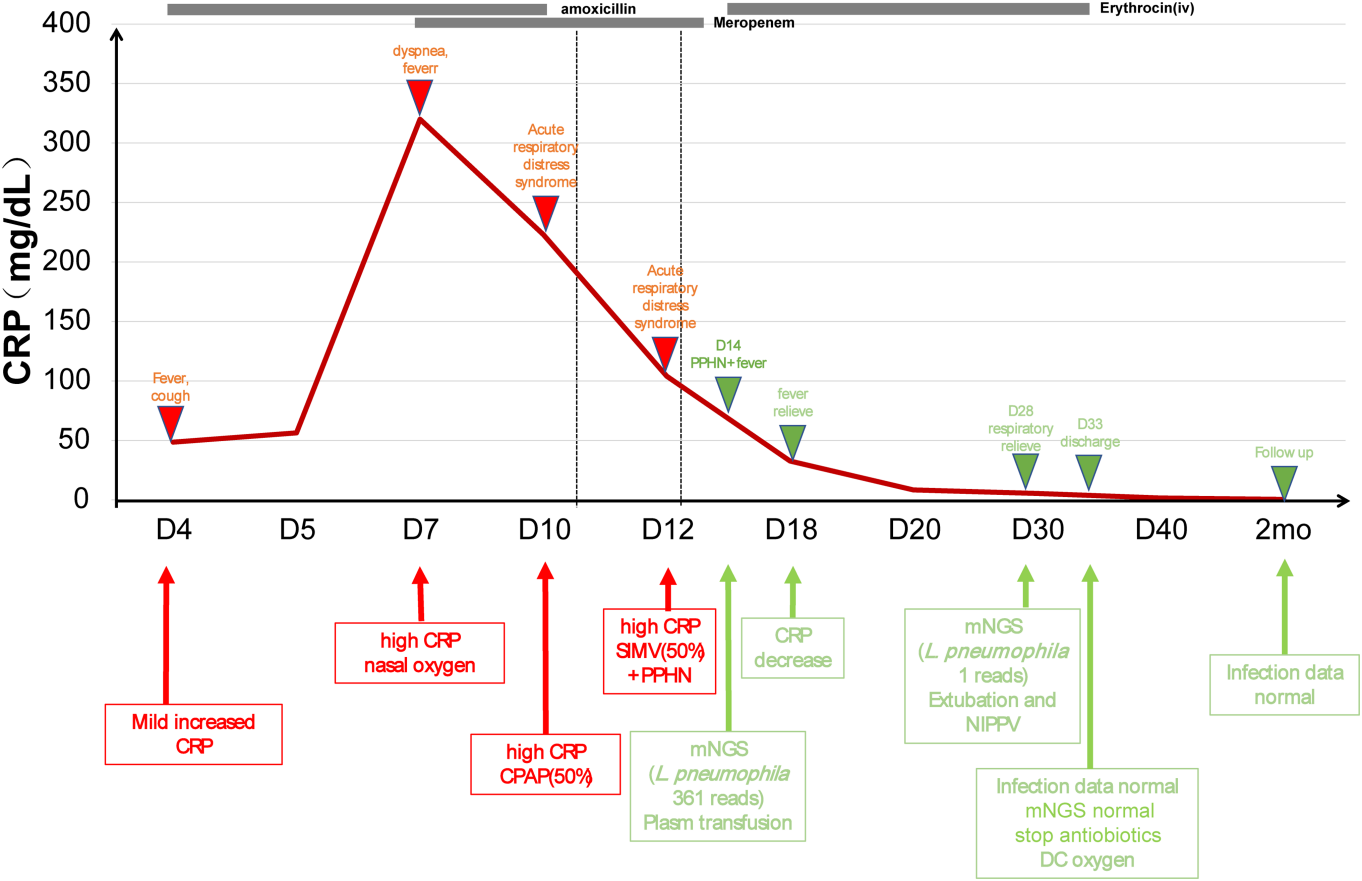
The treatment course in *Legionella pneumophila* case. CRP, C-reactive protein; NICU, neonatal intensive care unit; *L. pneumophila*, *Legionella pneumophila*.

Patient 2 (4-day-old female) was admitted to NICU due to high fever. The patient was born (3,400 g) *via* spontaneous pregnancy at 40 weeks to a 31-year-old mother (gravida 2, para 2). Apgar score was 10/10 at 1 and 5 min, respectively. After birth, the patient stayed at home for breast feeding using a circulation system. She developed fever and cough on day 4, which rapidly deteriorated. The patient had NCPAP support (35% oxygen) and persistent fever. The blood analysis showed a CRP higher than 320 mg/dl, PCT of 1.07 ng/ml, and lactate level of 4.3 mmol/l. On day 16, peripheral blood was collected for mNGS, which reported 361 reads of *Legionella pneumophila* while microbiological culture of sputum, blood, CSF, and BALF were negative. To confirm the mNGS findings, a BALF sample was collected and sent for 16s ribosomal RNA (rRNA) sequencing, which also identified *L. pneumophila* (Figure 3). In addition, the blood *Legionella pneumophila* IgM antibody was positive. We adjusted antibiotics to intravenous erythrocin and supported therapy (Figure 4) for 33 days. The patient was not discharged until mNGS of blood reported zero reads of *L. pneumophila*. The growth and neurological development were normal at 2-month follow-up.

#### Bacillus cereus

**Figure 5:**
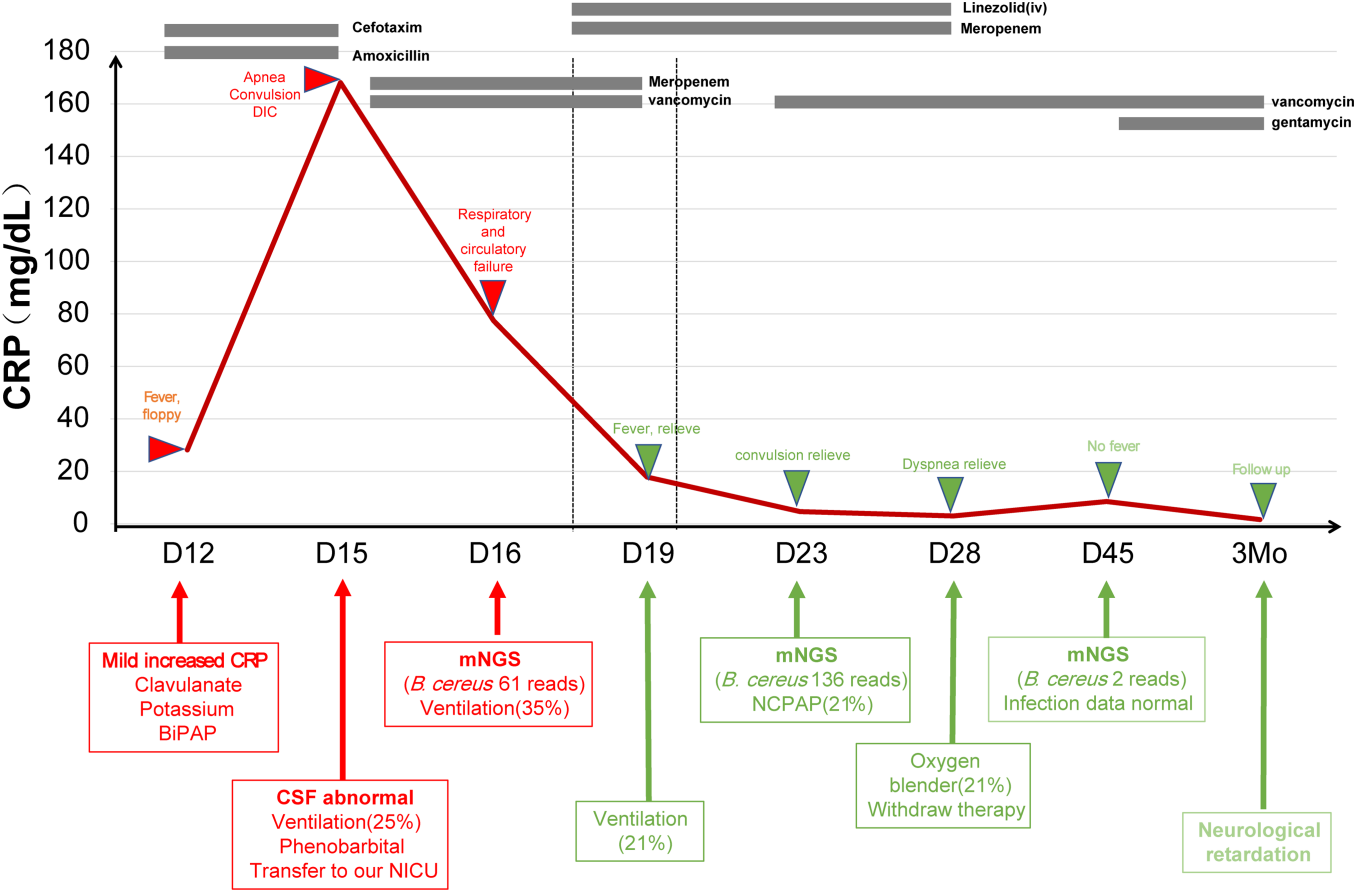
The treatment course in *Bacillus cereus* case. CRP, C-reactive protein; CSF, cerebral spinal fluid; NICU, neonatal intensive care unit; *B. cereus*, *Bacillus cereus*; and NCPAP, nasal continuous positive airway pressure.

Patient 10 (15-day-old female) presented with tachypnea for 15 days, fever for 3 days, and convulsion for 2 days. She was the younger twin born *via* caesarean section to a 34-year-old gravida 6, para 3 mother at 34^+2^ weeks weighed at 1,660 g. The patient was sent to the local neonatal ward for nasal continuous positive airway pressure treatment (NCPAP). She had fever (38°C) and was switched to BiBAP (Fi0_2_ 30%) on day 12. Intubation was performed due to apnea on day 14. Blood test reported a CRP of 168.1 mg/dl, PCT of 18.12 ng/ml. CSF test showed white blood cell of 9.65 × 10^9^/L, sugar of 1.02 mmol/L, and protein of 2.64 g/L. On day 16, CSF was collected and sent for mNGS, which reported 61 reads of *Bacillus cereus*. The same sample was also sent for 16s rRNA sequencing, which also showed *Bacillus* spp. Brain MRI revealed multiple encephalomalacia foci and venous thrombosis. According to one study (Lotte et al., 2017), we changed antibiotics to gentamicin and vancomycin (Figure 5). The patient was discharged from hospital on day 28 due to request of parents. The blood and CSF tests were normal during a follow-up visit two weeks later, but diffused encephalomalacia was still present.

## Discussion

According to the World Health Organization (WHO) reports in 2018, about 10,000 newborns worldwide were infected with *Mycobacterium tuberculosis*, of which women and children younger than 15 years old accounted for 32 and 11% of tuberculosis cases, respectively (WHO, 2019). Fetus infected with *Mycobacterium tuberculosis* intrauterine or during delivery is called congenital tuberculosis, which is a rare type but might leader to a high mortality rate (Jenkins et al., 2017). Literature reviewed reported no more than 400 cases in world limited treatment experience with *M. tuberculosis* infection (Newberry and Robertson Bell, 2018; Li et al., 2019; Shao et al., 2021). Congenital tuberculosis symptoms cover respiratory, neurologic, and gastrointestinal. The onset of symptoms in infants tends be quicker than in older children and adults. The sickest infants may develop respiratory failure, shock, disseminated intravascular coagulation, and multiple organ failure (Newberry and Robertson Bell, 2018). Congenital tuberculosis is difficult to diagnose due to atypical clinical manifestation (Solórzano Santos, 2015). In clinical practice, due to differences in individuals’ immune functions, the time of symptom onset varied significantly (Schaaf et al., 2010). Varik et al. (2012) reported the symptoms of congenital tuberculosis occurred within 3 weeks after birth, and the average age of onset was 28 days. In our case, the 19-day-old male presented fever and tachypnea as well as signs of respiratory failure. Even after mNGS finding, the *Mycobacterium tuberculosis* culture of peripheral blood, sputum, and BALF for 21 days was still negative. The etiological diagnosis has shortened the length of stay (mean duration of 36.5 days) and improved the patient’s prognosis (Li et al., 2019).

*Legionella pneumophila* can cause pneumonia, but rarely seen in neonates (Kleinberg and Antony, 2020). Molly et al. reported a 6-day-old neonate infected by *L. pneumophila*, which rapidly progressed to severe ARDS (Dorfman et al., 2020). The clinical presentations of Legionnaires’ disease in neonates included fever, cough, tachypnea, pneumonia in chest X-ray, acute respiratory distress, and sepsis. In our case, the patient presented with fever and progressive respiratory insufficiency that needed mechanical ventilation. When diagnosing legionellosis, samples from lower respiratory tract (sputum, BALF etc.) are preferred (Cattan et al., 2019) and the culture generally takes 3–5 days. The urine antigen test is also a first-line diagnostic test, although it is limited to the *Legionella pneumophila* serogroup 1 (Cunha et al., 2016). In our case, urine antigen and culture of the BALF were negative. The blood IgM antibody, BALF mNGS, and 16s rRNA sequencing were positive for *L. pneumophila*. Macrolide and quinolone antibiotics are suggested as first-line therapy of severe Legionella pneumonia (Cunha et al., 2016). For our patient, empirical antibiotics were first changed from amoxicillin to meropenem, neither of which was effective against *L. pneumophila*. Following the etiological diagnosis, erythrocin was administered with satisfying results.

Due to immature immune system and prolonged mechanical ventilation, neonates are particularly susceptible to disseminated infections caused by environmental organisms such as *B. cereus* (Schelonka and Infante, 1998). B. cereus is usually related to contaminated food and can lead to digestive manifestation and endophthalmitis (Lewin et al., 2019). Despite the small number of reported cases of *B. cereus* infection in infants, many of which were fatal. Twelve (75%) of the infants died, the majority of which occurred within 3 days of onset. Four infants who survived had developed cerebral palsy (Manickam et al., 2008). Imaging characteristics included hemorrhagic meningoencephalitis that first affected the white matter, and then the cortex and basal ganglia (Lequin et al., 2005). In our case, similar imaging features were observed in encephalomalacia foci in the white matter and then in the cortex and basal ganglia. The CSF, sputum, and blood culture were negative. But CSF mNGS and 16s rRNA sequencing were positive for *B. cereus*. Due to a beta-lactamase that renders it resistant to most penicillins and cephalosporins, effective antibiotics include chloramphenicol, clindamycin, vancomycin, erythromycin, and aminoglycosides (Drobniewski, 1993).

Our study has several limitations. First, this study was of a small sample size and was unable to sufficiently evaluate the clinical utility and drawbacks of mNGS in neonatal patients. Second, there are challenges associated with mNGS when applied in a clinical setting, such as the cost, complexity of the test, and lack of experimental standards. Third, the interpretation of results was difficult when distinguishing causal pathogens from colonizing or contaminating microorganisms. Last, all patients received empirical antibiotic treatment before mNGS was conducted, which might negatively impact the analytical sensitivity of detection microorganisms, despite that all cases returned positive findings.

## Data Availability Statement

The datasets presented in this study can be found in online repositories. The names of the repository/repositories and accession number(s) can be found at: https://www.ncbi.nlm.nih.gov/sra PRJNA808819.

## Ethics Statement

The studies involving human participants were reviewed and approved by Ethics Committee of Hunan Children’s Hospital (HCHLL-2021-71). Written informed consent to participate in this study was provided by the participants’ legal guardian/next of kin.

## Author Contributions

RZ, X-mP, and CL conceptualized and designed this study. RZ and YZ drafted the paper. Z-hX and C-yL conducted statistical analysis on the data. FZ, W-qH, and MZ gave a lot of assistance and revised manuscript. All authors contributed to the article and approved the submitted version.

## Funding

This work was supported by Natural Science Foundation of Hunan Province, China (grant no. 2021JJ40278).

## Conflict of Interest

CL and C-yL were employed by Hangzhou Matridx Biotechnology Co., Ltd.

The remaining authors declare that the research was conducted in the absence of any commercial or financial relationships that could be construed as a potential conflict of interest.

## Publisher’s Note

All claims expressed in this article are solely those of the authors and do not necessarily represent those of their affiliated organizations, or those of the publisher, the editors and the reviewers. Any product that may be evaluated in this article, or claim that may be made by its manufacturer, is not guaranteed or endorsed by the publisher.

## Footnotes

^1^ http://bio-bwa.sourceforge.net
